# Strain-Specific Variability in Viral Kinetics, Cytokine Response, and Cellular Damage in Air–Liquid Cultures of Human Nasal Organoids After Infection with SARS-CoV-2

**DOI:** 10.3390/v17101343

**Published:** 2025-10-06

**Authors:** Gina M. Aloisio, Trevor J. McBride, Letisha Aideyan, Emily M. Schultz, Ashley M. Murray, Anubama Rajan, Erin G. Nicholson, David Henke, Laura Ferlic-Stark, Amal Kambal, Hannah L. Johnson, Elina A. Mosa, Fabio Stossi, Sarah E. Blutt, Pedro A. Piedra, Vasanthi Avadhanula

**Affiliations:** 1 Department of Molecular Virology and Microbiology, Baylor College of Medicine, Houston, TX 77030, USA; gina.aloisio@bcm.edu (G.M.A.);; 2 Department of Pediatrics, Baylor College of Medicine, Houston, TX 77030, USA; 3 Advanced Technology Cores, Baylor College of Medicine, Houston, TX 77030, USA; 4 Department of Molecular and Cellular Biology, Baylor College of Medicine, Houston, TX 77030, USA

**Keywords:** SARS-CoV-2, airway organoids, variants, host responses

## Abstract

SARS-CoV-2 variants have demonstrated distinct epidemiological patterns and clinical presentations throughout the COVID-19 pandemic. Understanding variant-specific differences at the respiratory epithelium is crucial for understanding their pathogenesis. Here, we utilized human nasal organoid air–liquid interface (HNO-ALI) cell cultures to compare the viral replication kinetics, innate immune response, and epithelial damage of six different strains of SARS-CoV-2 (B.1.2, WA, Alpha, Beta, Delta, and Omicron). All variants replicated efficiently in HNO-ALIs, but with distinct replication kinetic patterns. The Delta variant exhibited delayed replication kinetics, achieving a steady state at 6 days post-infection compared to 3 days for other variants. Cytokine analysis revealed robust pro-inflammatory and chemoattractant responses (IL-6, IL-8, IP-10, CXCL9, and CXCL11) in WA1, Alpha, Beta, and Omicron infections, while Delta significantly dampened the innate immune response, with no significant induction of IL-6, IP-10, CXCL9, or CXCL11. Immunofluorescence and H&E analysis showed that all variants caused significant ciliary damage, though WA1 and Delta demonstrated less destruction at early time points (3 days post-infection). Together, these data show that, in our HNO-ALI model, the Delta variant employs a distinct “stealth” strategy characterized by delayed replication kinetics and epithelial cell innate immune evasion when compared to other variants of SARS-CoV-2, potentially explaining a mechanism that the Delta variant can use for its enhanced transmissibility and virulence observed clinically. Our findings demonstrate that variant-specific differences at the respiratory epithelium could explain some of the distinct clinical presentations and highlight the utility of the HNO-ALI system for the rapid assessment of emerging variants.

## 1. Introduction

The coronavirus disease 2019 (COVID-19) pandemic, caused by severe acute respiratory syndrome coronavirus 2 (SARS-CoV-2), has resulted in hundreds of millions of confirmed infections worldwide, and more than seven million deaths worldwide, though this is likely an underestimate [1]. SARS-CoV-2 infection often results in an asymptomatic infection or mild illness but can also lead to severe disease including respiratory failure and death, particularly in the elderly and individuals with underlying medical conditions [2].

SARS-CoV-2 predominantly infects the epithelial lining of both the upper and lower respiratory tracts by binding the spike protein to the angiotensin-converting Enzyme 2 (ACE2) receptor. The spike protein or the attachment protein of SARS-CoV-2 is the site of several mutations which enhance its binding, and has evolved into multiple variants showing enhanced transmissibility or immune evasion [3]. Variants of SARS-CoV-2 were classified based on their potential impact on public health. The World Health Organization (WHO) designates variants of concern (VOCs) when they show evidence of an increased transmissibility, more severe disease, reduced effectiveness of treatments or vaccines, or diagnostic detection failures [4].

One of the first COVID-19 infections in the United States was in Washington State, recorded as strain USA/WA1/2020 [5]. The Alpha variant was identified in the UK in 2020, which had an estimated 40–70% higher transmission from other circulating SARS-CoV-2 strains [6]. Next was the Beta variant, again noted to have an increased transmission over the WA1 strain [7]. Delta was first detected in late 2020 in India, but by 2021 had become the predominate variant globally. This variant was characterized by an even higher transmissibility. Despite the increased number of cases, the Delta variant had a lower fatality rate than prior variants, attributed to vaccine usage [8]. However, when only examining unvaccinated patients, studies reported a higher morbidity and mortality [9]. Finally, Omicron emerged in Botswana in 2021 and was characterized by immune evasiveness and resistance to prior vaccines, but overall milder clinical symptoms than other variants [10,11,12,13].

Several studies have provided valuable insights into the differences in the biological behavior of SARS-CoV-2 variants. One of the first studies used human pluripotent stem cell-derived organoids to study SARS-CoV-2’s ability to infect multiple organs, including the pancreas, liver, heart, and brain [14]. Another study demonstrated that the Delta strain had a higher replication efficiency than the Alpha variant in both airway organoids and human airway epithelial cells [15]. We and others have developed a human nasal organoid air–liquid interface (HNO-ALI) system derived from nasal (tissue) stem cells that successfully modeled SARS-CoV-2 infection [16,17]. Chiu et al. went on further to confirm the higher infectivity of the Delta and Omicron variants in differentiated nasal organoid monolayers [17]. Other studies observed that Delta displayed an enhanced replication in lung organoids compared to ancestral strains or in differentiated human nasal epithelial cultures compared to the Beta variant [18], while Shuai et al. found that Omicron exhibited a reduced pathogenicity in human broncheoalveolar organoids despite maintaining a high infectivity [19,20]. Interestingly, Suzuki et al. found that, while Omicron replicated efficiently in upper respiratory tract cell models, it showed an attenuated replication in lung cell cultures, potentially explaining its milder clinical presentation [21]. Other groups using iPSC-derived alveolar and airway cells in micro-patterned culture plates showed that the Delta variant proliferated the most actively among the SARS-CoV-2 variants [22]. An in-depth study on various Omicron variants demonstrated that, of all Omicron variants, BA.5 showed a higher infectivity than the parent Omicron B.1.1.529 variant [23]. In our previous studies, we described the development of HNO-ALIs using non-invasive methods such as nasal swabs and nasal wash [24]. Our HNO-ALI cultures mimic the nasal epithelium of the upper respiratory tract containing multiple cell types such as ciliated cells, goblet cells, club cells, and basal cells. In addition, the HNO-ALI lines derived from different individuals elicit distinct innate immune responses and show age-specific characteristics [24,25].

In the present study, we sought to compare viral kinetics, innate epithelial cytokine responses, and cellular injury between six strains of SARS-CoV-2 using our HNO-ALI system as an ex vivo human challenge model system. We found that, compared to other strains, the Delta variant takes longer to achieve maximum viral growth, poorly elicits the epithelial innate immune response, and causes less damage to the apical ciliated cells. Overall, we propose that the Delta variant has a unique “stealth” strategy, employing delayed viral kinetics and a dampened immune activation to evade host anti-viral responses in our HNO-ALI system, which could potentially explain some of the Delta strain’s increased transmissibility and clinical virulence.

## 2. Methods

### 2.1. HNO-ALI Cell Lines

Human nose organoid HNO-ALI cell lines were obtained from Baylor College of Medicine 3D Organoid Core and cultured using standard conditions as described [14]. Briefly, nasal washes and swabs were collected from two females between the ages of 25–50 after obtaining signed informed consent under an approved protocol by Baylor College of Medicine Institutional Review Board. Samples were placed in digestion media [10 mL airway organoid (AO) medium + 10 mg Collagenase (Sigma C9407) + 100 μL Amphotericin B], strained to remove debris, and washed several times. After pelleting of the cells and removal of the supernatant, cells were suspended in Matrigel^®^ (Corning, New York, NY, USA) and plated for cell expansion as three-dimensional (3D) HNOs for approximately 5–7 days in growth media. HNOs were then enzymatically and mechanically sheared in order to make a single cell suspension, and seeded onto transwells^®^ (Corning, New York, NY, USA) at a density of 3 × 10^5^ cells/well. HNO-ALIs were maintained in AO media with endothelial growth factor (EGF) (Peprotech-AF-100-15) containing 10 μM Y-27632. After 4 days, the monolayers of cells were subsequently maintained in an air–liquid environment with differentiation media (PneumaCult-ALI medium^®^ from STEMCELL Technologies, Cambridge, MA, USA) added to the lower (basolateral) compartment of the transwells^®^, and the epithelium (apical compartment) was air-exposed and maintained in a humidified incubator at 37 °C with 5% CO_2_. The differentiation media was replaced every 4 to 5 days. HNO-ALI cultures were maintained for a total of 21 days, during which differentiation occurred into a pseudostratified multi-cellular ciliated epithelium. At the end of 21 days of differentiation in an air–liquid environment, the HNO-ALI cultures were used for the viral infection studies.

### 2.2. Virus Stocks

Isolate hCoV-19/USA-WA1/2020, NR-52281 (SARS-CoV-2 USA_WA1/2020) was obtained through BEI Resources NIAID, NIH, and deposited by the Centers for Disease Control and Prevention (Viral Sequence deposited at https://www.ncbi.nlm.nih.gov/nuccore/MN985325.1, accessed on 1 October 2025), and SARS-CoV-2 USA/MD-HP01542/2021 (Lineage B.1.351; Beta Variant) NR 55282 was obtained through BEI Resources NIAID, NIH, in Homo sapiens Lung Adenocarcinoma (Calu-3) Cells, NR-55282, contributed by Andrew S. Pekosz (Sequence deposited at GISAID: EPI_ISL_890360).

SARS-CoV-2, Alpha, Delta, and Omicron strains were isolated from nasal wash samples of patients (viral sequences of clinical isolates are submitted as Supplementary Materials). Nasal swab sample collection was performed under Baylor College of Medicine institutional review board-approved protocol with waiver of consent. Clinical isolates were cultured in Vero E6 cells in MEM supplemented with 10% FBS, 10,000 U/mL penicillin/10,000 µg/mL streptomycin/25 μg/mL amphotericin B, l-glutamine (200 mM). For virus propagation, Vero E6 cells were seeded in T-150 flasks and incubated for 24–48 h until 75–90% confluency at 36 °C with 5% CO_2_. Cells were then infected with SARS-CoV-2 variants at a multiplicity of infection (MOI) of 0.01 with media containing 2% FBS-MEM and incubated for 3–4 days. Virus was harvested when 50–75% of CPE was observed. SARS-CoV-2 harvest was sonicated and centrifuged, and supernatant was mixed with Iscove’s Modified Dulbecco’s Medium/15% glycerol at 1:1 ratio. One ml aliquots of SARS-CoV-2 stocks were snap-frozen on dry-ice alcohol baths and stored at −80 °C.

### 2.3. Study Design

Two adult HNO-ALI cell lines (HNO-ALI 919 and HNO-ALI 920) were used for this study. The differentiated HNO-ALI cultures were apically infected with SARS-CoV-2 (WA1, B.1.2, Alpha, Beta, Delta, or Omicron strains) at a 0.01 MOI. Two dedicated and independent technical replicates of infected HNO-ALI transwells^®^ were used for measuring outcomes at each time point, 1, 3, and 6 dpi, totaling to six transwells per HNO-ALI line per viral condition. The outcomes measured from the apical and basolateral supernatant samples were virus gene copy numbers and cytokines and chemokines, while the membrane-attached epithelium was processed for cell composition and cytopathology.

### 2.4. Sample Collection

Apical wash samples were collected using three consecutive 200 µL washes with AO differentiation media. The combined 600 µL apical washes were diluted 1:1 with 15% glycerol/Iscove media; aliquots were prepared, snapped-frozen, and stored at −80 °C. All 600 µL samples of the basolateral media were collected and diluted 1:1 with 15% glycerol/Iscove’s media; aliquots were prepared, snapped-frozen, and stored at −80 °C.

### 2.5. Viral Infection

All work with SARS-CoV-2 was performed in a class II biosafety cabinet in the biosafety level 3 (BSL-3) high-containment facility at BCM. HNO-ALI cell lines were infected with SARS-CoV-2 strains at an MOI of 0.01. Briefly, the virus inoculum (30 µL/well) of WA1, B.1.2, Alpha, Beta, Delta, or Omicron was added to the apical compartment for 1.5 h at 36 °C with 5% CO2, and then the inoculum was removed. For mock infection, AO differentiation media (30 µL/well) alone was added, incubated for 1.5 h, and then removed.

### 2.6. Quantitative PCR Assay

The viral RNA was extracted using a mini viral RNA kit (Qiagen Sciences) in an automated QIAcube platform according to the manufacturer’s instructions [26]. Viral RNA was detected and quantified (virus gene copy number) using real time polymerase chain reaction (RT-PCR) with primers targeting the nucleocapsid genes of SARS-CoV-2 as previously described [26].

### 2.7. Cytokine and Chemokine Assay

Cytokines and chemokines secreted by HNO-ALIs in the apical and basolateral compartments were measured and analyzed using the Milliplex cytokine/chemokine magnetic bead panel (Millipore, Burlington, MA, USA) according to the manufacturer’s instructions. The kits used in this study include (i) the Milliplex human cytokine panel with eotaxin/CCL11, fibroblast growth factor 2 (FGF-2), granulocyte colony stimulating factor (G-CSF), granulocyte–macrophage colony stimulating factor (GM-CSF), interleukin 1 alpha (IL-1α), interleukin 1 beta (IL-1β), interleukin 6 (IL-6), interleukin 8 (IL-8/CXCL8), interleukin 17E (IL-17E/IL-25), interferon gamma-induced protein 10 (IP-10/CXCL10), monocyte chemoattractant protein 1 (MCP-1), monocyte chemoattractant protein 3 (MCP-3), monokine induced by gamma interferon (MIG/CXCL9), macrophage inflammatory protein 1 alpha (MIP1α), macrophage inflammatory protein 1 beta (MIP1β), regulated on activation, normal T cell expressed and secreted (RANTES/CCL5), tumor necrosis factor alpha (TNFα), vascular endothelial growth factor a (VEGF-A), interleukin 33 (IL-33), interferon gamma inducible T-cell alpha chemoattractant (TAC/CXCL11), and interleukin 29 or interferon lambda 1 (IL-29/IFN-λ); (ii) the transforming growth factor beta (TGFβ1 Singleplex kit); (iii) the Milliplex human MMP panel 2 with matrix metallopeptidase 9 (MMP9) and matrix metallopeptidase 7 (MMP7); and (iv) the Milliplex human tissue inhibitor of metalloproteinases (TIMP) panel 2 with TIMP1. Data were obtained with Luminex xPONENT for MAGPIX v4.2 build 1324 and analyzed with MILLIPLEX Analyst v5.1.0.0 standard build. All cytokine concentrations less than the lowest standard of each analyte are considered negative, and a value half the concentration of the lowest standard was imputed.

### 2.8. Immunohistochemistry (IHC) and Immunofluorescence Staining

HNO-ALI cell lines were fixed in image-iT™ Fixative Solution (4% formaldehyde) [Catalog number: FB002] for 15 min, followed by dehydration in ethanol series (30%, 50%, and 70%, each 30 min at room temperature or overnight at 4 °C). The transwell^®^ membranes were then embedded in paraffin and sectioned. Standard hematoxylin and eosin (H&E) staining was performed. For immunofluorescence staining, the sections were deparaffinized in Histo-Clear^®^, followed by washes in an alcohol sequence (100 > 100 > 90 > 70%). Then, the slides were rehydrated and exposed to heat-induced antigen retrieval in 10 mM citrate buffer pH [27]. The sections were then washed in water for 5 min and blocked for 30 min in 2% bovine serum albumin (BSA) in blocking buffer (PBS). The sections were incubated overnight at 4 °C with the following primary antibodies: keratin 5 (KRT5) for basal cells (1:2000 BioLegend, San Diego, CA, USA, Catalog number: 905503); acetylated alpha tubulin for cilia (1:1000, Santa Cruz, TX, USA, sc-23950); Mucin 5AC for goblet cells (1:1000, Invitrogen, Carlsbad, CA, USA, Catalog number: 45M1); and guinea pig polyclonal antibody specific for SARS-CoV-2 (1:2,000, BEI Resources, Manassas, VA, USA, catalog no. NR10361). Primary antibodies were washed three times in PBS for 10 min each, incubated with secondary antibodies (donkey anti-mouse 488 Invitrogen, A-21202, donkey anti-rabbit 568, Invitrogen, A-10042, goat anti-guinea pig 647, Invitrogen, A-21450) for 2 h at room temperature, washed three times with PBS, stained with 4′,6-diamidino-2-phenylindole (DAPI), washed twice with PBS, and mounted in Vectashield Plus^®^ (Vector Laboratories, Newark, CA, USA Catalog number H-1900). The slides were stored at −20 °C.

### 2.9. Immunofluorescence Image Quantification and Analysis

Samples were imaged using high-resolution Cytiva DVLive (Cytiva, Marlborough, MA, USA) or Olympus IX83 epifluorescence deconvolution microscopes (Olympus, Tokyo, Japan). Images were collected with both a 20×/0.75NA and a 60×/1.42NA objective lens, with a 10 µm z-stack (using optical sections at the recommended Nyquist for each objective). Three-dimensional images were deconvolved using a quantitative image restoration algorithm.

Max intensity projections were used for image analysis and processed using Fiji [28]. Each experiment had a minimum of three different fields of view quantified as cell counts in Fiji. The average cell count of the different fields was obtained for each specific cell type at the midpoint (3 dpi) and the endpoint (6 dpi) of infection assays. Counts of goblet cells (Muc5AC+ stained cells) and basal cells (Krt5+ stained cells) after infection with SARS-CoV-2 were quantified as percentages relative to the total number of DAPI+ cells.

For epithelial area, the epithelium was manually outlined using the draw setting in Fiji and quantified by using the “calculate area” function. The approximate area of apical ciliated cells (acetylated alpha tubulin +) and mucous (Muc5AC+) was quantified using Fiji particle analysis to calculate a total area in pixels. Fluorescence thresholding was normalized to mock infection. The epithelial area, mucous area, and ciliated area were then converted to μm using the pixel size information from the imaging specs.

### 2.10. Sex as a Biological Variable

Sex was not considered as a biological variable.

### 2.11. Statistical Analysis

The study was designed to determine if there were differences in cellular response between strains of SARS-CoV-2. Study factors were infection condition, time, and cell surface. Outcome variables measured were virus replication, cytokine expression, and cell composition.

For each outcome variable (except where noted below), a 7 × 2 × 3 factorial design approach characterized by infecting condition (mock, WA1, B.1.2, Alpha, Beta, Delta, and Omicron), cellular compartment (apical or basolateral), and time (1, 3, and 6 dpi) was undertaken to analyze the differences between strains of SARS-CoV-2. Duplicate response values were obtained for most data points; therefore, a single observation was represented by the average of the technical replicate response values for analyses. Time was treated as a continuous factor to evaluate linear and quadratic effects. Strain of SARS-CoV-2 was included as a variable of primary interest.

All two-way interactions with infecting condition (SARS-CoV-2 strain), cell surface, and time (dpi) were included in the model to test the effects of viral replication and viral expression by cell surface. Highly non-significant interaction terms were dropped one at a time from the model to re-evaluate the model with the remaining interaction terms; variables were retained in the model if it was shown that there was some indication of significance (a threshold of *p* < 0.20). For a significant interaction term, the emmeans package (1.8.7) with the contrast function was used to generate contrast tests for comparing the difference in levels of one factor (either infection condition or cell surface) with each of the levels in the other factor. No multiple comparison adjustment procedure was applied. Point estimates (beta coefficients, odds ratios, relative risks) were reported, along with the corresponding 95% confidence interval (CI), and statistical significance was assigned when *p* values were <0.05. Statistical analyses were performed with R software v4.3.2 (R Foundation for Statistical Computing).

Virus replication: Ordinary least squares linear regression was used to analyze viral kinetics of SARS-CoV-2 strains WA1, B.1.2, Alpha, Beta, Delta, and Omicron, as measured by PCR log_10_ copy numbers from the apical and basolateral wash samples, over three time points.

Cytokine expression: We evaluated 24 different cytokines in both the apical and basolateral compartments from two different HNO-ALIs infected with SARS-CoV-2 strains WA1, B.1.2, Alpha, Beta, Delta, Omicron, or mock over three time points. To account for the differing magnitudes and variances of the cytokine responses, the raw cytokine concentration values were log transformed and then standardized by converting to Z-scores. For each cytokine, a Z-score was calculated by subtracting the overall average concentration from the individual concentration value and dividing that result by the standard deviation of all concentration values within that single cytokine. A composite Z-score was calculated for each cytokine functional group by taking the average of the Z-scores of the respective cytokines within a cytokine group. These composite Z-scores were then converted into probabilities from a standard normal cumulative distribution function using the pnorm function in R. A separate fractional logit regression was performed on each cytokine functional group’s composite Z-score probabilities based on a quasi-binomial generalized linear model. Fractional logit regression implements quasi-maximum likelihood estimators with robust standard errors fitting a model conditional on the set of independent variables (infection condition, cell compartment, HNO line, time, and 2-way interactions) providing estimates in odds ratios (OR).

Cell composition: Lastly, we quantitated the percent of basal and goblet cells, as well as cilia, mucous, and epithelial area in the HNO-ALIs over time, describing cell type specific changes during SARS-CoV-2 infection. Percentages of basal and goblet cells were rescaled to the range 0–1 by dividing by 100 for use in fractional logit regression. Poisson regression analysis was performed to analyze cilia, mucous, and epithelial area to obtain robust standard errors for the risk ratio (RR) estimates.

## 3. Results

### 3.1. Delta Reaches a Steady State Later than Other SARS-CoV-2 Variants

**Figure 1:**
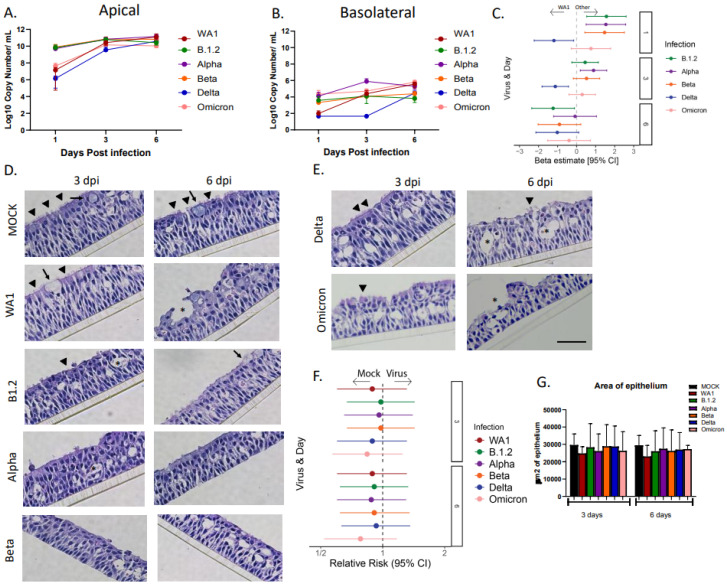
**Replication kinetics and morphologic analysis of SARS-CoV-2 infected HNO-ALIs.** (**A**) Apical log_10_ viral gene copy numbers for two HNO-ALIs for strains Washington, B.1.2, Alpha, Beta, Delta, and Omicron, (**B**) corresponding to basolateral log_10_ viral gene copy numbers. Error bars (standard deviation) represent two technical replicates of two donor HNO-ALIs. (**C**). Forest plot demonstrating the adjusted beta coefficient estimate with 95% confidence intervals, represented by dots and T-bars, respectively, between strains of SARS-CoV-2 by days post infection (dpi). (**D**,**E**) H&E images of single adult HNO-ALI line at 3 and 6 dpi with six strains of SARS-CoV-2. Scale bar is 100 µm. Black inverted triangles indicate cilia, arrows indicate goblet cells, and asterisks indicate cell damage. (**F**) Comparison of epithelial area of HNO-ALIs between strains of SARS-CoV-2 or mock infection. (**G**) Area of epithelium of six strains of SARS-CoV-2 at 3 and 6 dpi. Error bars (standard deviation) represent two technical replicates of two donor HNO-ALIs.

To determine differences in viral kinetics, viral gene copy levels were compared between variants of SARS-CoV-2; two HNO-ALIs derived from two patients were infected with six different strains of SARS-CoV-2 (B.1.2—the predominant strain circulating in Houston, Texas, during 2020, WA1, Alpha, Beta, Delta, and Omicron). While all SARS-CoV-2 variants replicated well and were detected on the apical side, there were substantial differences in the viral gene copy levels. Compared to the original WA1 variant, the B.1.2, Alpha, and Beta strains were detected apically at higher viral gene copy numbers at day 1 post infection (1 day(s) post infection (dpi)). By mid-infection (3 dpi), WA1 and Omicron had reached comparable viral gene copy number production to these strains, with five of six strains (WA1, B.1.2, Alpha, Beta, and Omicron) achieving a steady state at peak infection (9–10 × 10^7^ viral gene copies/mL). Interestingly, the Delta strain lagged behind the other variants, achieving a peak state at 6 dpi (Figure 1A). Viral RNA was also detected basolaterally, suggesting damage to the epithelium or replication of the virus in cell types other than apical ciliated cells. Viral RNA was detected in the basolateral compartment in four of six strains at 1, 3, and 6 dpi (steady state 4–6 × 10^7^ viral gene copies/mL). However, the Delta variant again lagged behind other strains, and was below the level of detection at 1 and 3 dpi.

We then used regression to compare viral gene copy numbers both in the apical and basolateral compartments. As WA1 was the original strain in the COVID-19 outbreak, we used this strain as a reference. The Delta strain of SARS-CoV-2 had a significantly lower detectable viral gene copy number at 1 dpi (Beta estimate: −1.19; 95% CI: −2.23, −0.15) and 3 dpi (Beta estimate: −1.12; 95% CI: −1.82, −0.43). However, by 6 dpi, the amount of Delta viral RNA was similar to the other strains. No other variants were significantly different to the WA1 strain at any time point (Figure 1C). H&E analysis of the HNO-ALIs infected with SARS-CoV-2 demonstrated a loss of apical ciliated cells and disruption of epithelial architecture compared to the mock infection (Figure 1D,E). WA1 and Delta strains appeared to be less destructive than other strains at day 3, with intact cilia, although less than the mock infected cells (Figure 1D,E). There was no difference in the epithelial area with regard to the viral infection or time (Figure 1F,G).

### 3.2. The Majority of the SARS-CoV-2 Variants Except Delta Induced Robust Cytokine and Chemokine Responses

**Figure 2:**
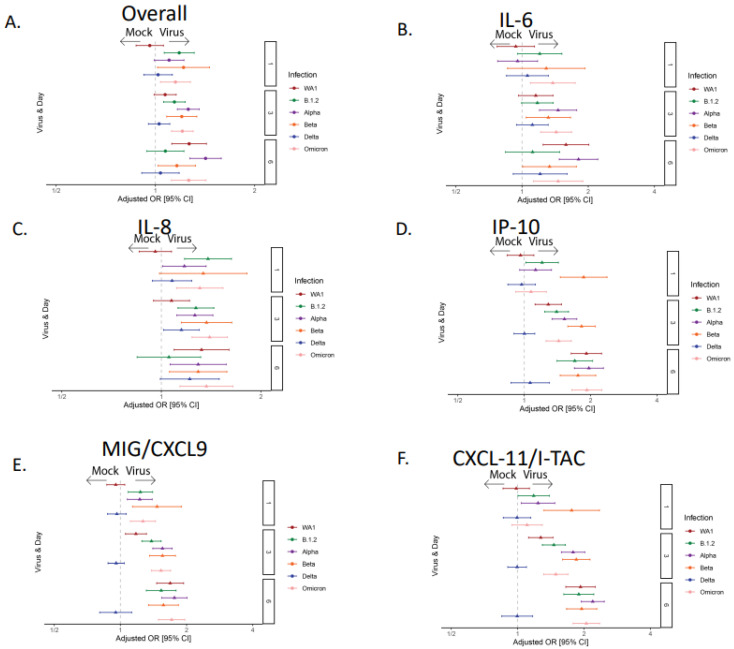
**Epithelial cytokine and chemokine response during infection with different strains of SARS-CoV-2.** Cytokine/chemokine secretion was modeled on the following factors: infection condition, cell line, cell surface, and dpi. Adjusted odds ratio estimates and their associated 95% confidence intervals are represented by dots and T-bars, respectively. (**A**) Forest plot showing the adjusted odds ratio with 95% confidence interval for overall cytokine secretion between strains of SARS-CoV-2. (**B**) Forest plot showing the adjusted odds ratio with 95% confidence interval cytokine expression of IL-6, (**C**) IL-8, (**D**) IP-10, (**E**) CXCL9, and (**F**) CXCL11.

To characterize epithelial-specific changes in cytokine secretion during SARS-CoV-2 infection, we performed a 24 cytokine Luminex analysis at 1, 3, and 6 dpi (Supplementary File S2). The differences in the epithelial cytokine response in HNO-ALIs both at baseline and during infection with SARS-CoV-2 variants were analyzed using composite Z-score probabilities. When examining all cytokines grouped together, the WA1, Alpha, Beta, and Omicron variants had a significantly higher overall cytokine expression at 6 dpi compared to the mock infection (Figure 2A). Strain B.1.2 had a significantly higher cytokine expression than the mock infection only at 3 dpi (Figure 2A). Cytokines classically induced in human infections with SARS-CoV-2, such as IL-6, IL-8, and IP-10, were all induced by all variants, demonstrating the fidelity of the HNO-ALI model. Interestingly, the Delta strain did not significantly induce IL-6 or IP-10, and only modestly significantly induced IL-8 (Figure 2B–D). Additionally, we found that CXCL9 and CXCL11, chemokines both up regulated in bronchoalveolar lavage fluids from patients with COVID-19 [29,30], increased during infection in all variants except Delta. Tumor necrosis factor alpha (TNFα) did not significantly change after infection with SARS-CoV-2 variants (Supplementary Files S1 and S2), potentially due to a lack of circulating immune cells.

**Figure 3:**
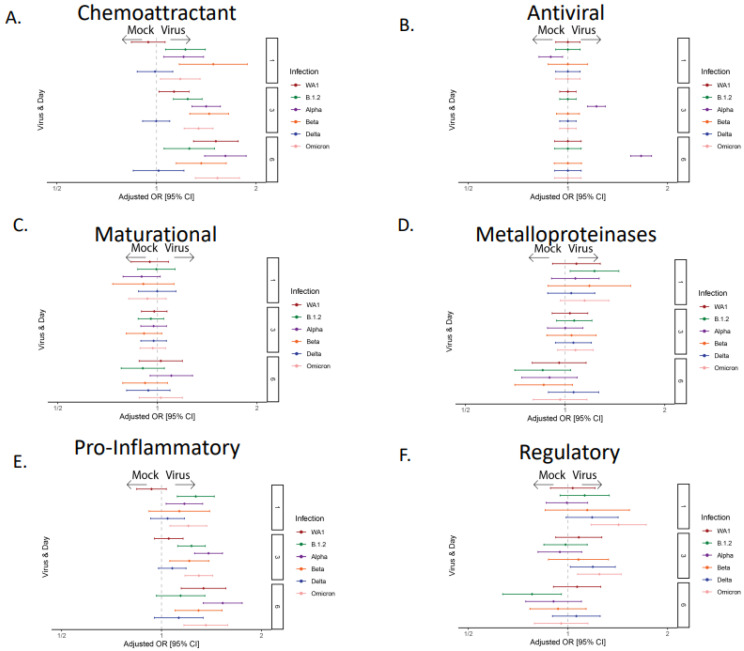
**Epithelial cytokine and chemokine groups during infection with different strains of SARS-CoV-2.** Forest plot showing the adjusted odds ratio with 95% confidence interval for grouped cytokine secretion between strains of SARS-CoV-2. Groups include (**A**) chemoattractant, (**B**) anti-viral, (**C**) maturational, (**D**) metalloproteinases, (**E**) pro-inflammatory, and (**F**) regulatory cytokines.

Cytokines were grouped by their biological function, which included chemoattractant (MCP-1, MCP-3, MIP-1α, MIP-1β, MIG/CXCL-9, IP-10, eotaxin, and RANTES), anti-viral (IL-29), maturational (VEGF-α, FGF-2, G-CSF, and GM-CSF), metalloproteinase and its inhibitor (MMP-7, MMP-9, and TIMP), pro-inflammatory (IL-1α, IL-1β, TNFα, IL-6, and IL-8), and regulatory (TGF-β) cytokines. The expression of these groups was compared across all six SARS-CoV-2 variants. Consistent with other studies, the chemoattractant and pro-inflammatory cytokine groups were the most highly secreted across SARS-CoV-2 variants [31,32,33,34,35], although Delta again was the exception, with no significant increase compared to the mock infection (Figure 3A–F). Interestingly, we found that only the Alpha strain of SARS-CoV-2 elicited a significant anti-viral detection, driven by IL-29 (interferon-lambda), an epithelial-derived interferon.

**Figure 4:**
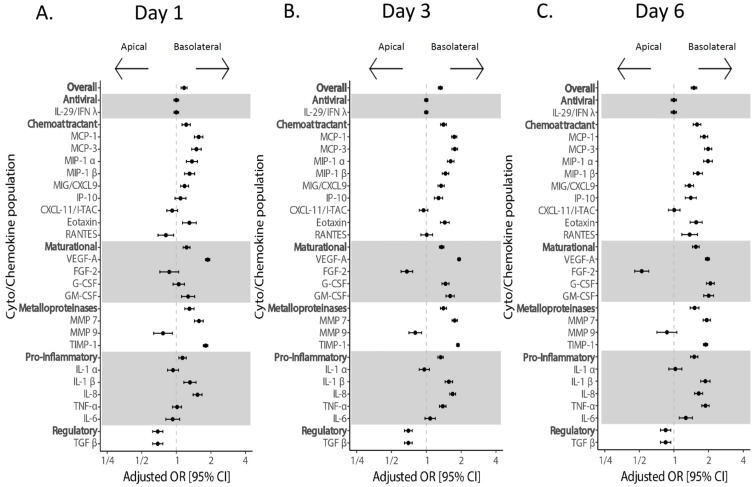
**Location of cytokine and chemokine release over time.** Cytokine/chemokine secretion was modeled on the following factors: infection condition, cell line, cell surface, and dpi. Forest plot with adjusted odds ratio with 95% confidence intervals of apical or basolateral expression of cytokines. Individual cytokines are listed below their respective groups. Adjusted odds ratio estimates and their associated 95% confidence intervals are represented by dots and T-bars, respectively. Time points are as follows: (**A**) 1 dpi. (**B**) 3 dpi. (**C**) 6 dpi.

We subsequently examined the apical vs. basolateral secretion of cytokines, which would mimic a release into the airway versus circulation, respectively (Figure 4). We found an overall higher secretion of cytokines in the basolateral compartment as the infection progressed. Every functional group had significantly higher levels in the basolateral compartment except for the regulatory group, which was significantly higher in the apical compartment, and anti-viral group, which was not different between the two compartments. In addition, FGF-2 and MMP-9 were detected at higher concentrations in the apical compartment.

When examining individual cytokines, the chemokines IP10, CXCL11, and CXCL9 (involved in the recruitment and maturation of the immune response) were upregulated after infection with all strains of SARS-CoV-2 except Delta. IP-10 and CXCL9 were detected at a significantly higher concentration in the basolateral compartment (Figure 4), even though the majority of SARS-CoV-2 RNA was detected on the apical side. The expression of maturational factors did not increase during infection (Figure 3), though there were several differences in location, with FGF-2 located apically, and others such as VEGF and G-CSF in the basolateral compartment (Figure 4). Pro-inflammatory cytokines, with the exception of the Delta strain, increased with infection (Figure 3) and were found predominantly in the basolateral compartment (Figure 4). Exceptions to this were IL1α, which favored the apical compartment, and IL-6, which changed from being expressed in both compartments to basolateral during late infection (6 dpi). Matrix metalloproteinase expression did not change with infection (Figure 3), potentially due to inhibition by TIMP-1. While MMP7 and TIMP-1 were basolateral, MMP9 was expressed apically. Regulatory cytokine (TGF-β) was significantly increased in early infection (1 and 3 dpi) in Omicron and Delta strains and appeared to be suppressed late in the infection for B.1.2.

Taken together, this suggests that, in our HNO-ALI model, the WA1, Alpha, B.1.2, Beta, and Omicron strains elicit a higher overall cytokine response during infection, predominantly in the pro-inflammatory and chemoattractant groups, than the Delta strain. The fact that certain cytokines such as CXCL9, IP-10, and CXCL11 remain unchanged in the Delta infection argues against the dampened immune response being solely due to the viral copy number. This could be further dampened by the increased expression of regulatory cytokines early in the infection.

### 3.3. Infection with SARS-CoV-2 Variants Causes Significant Ciliary Damage in HNO-ALIs

**Figure 5:**
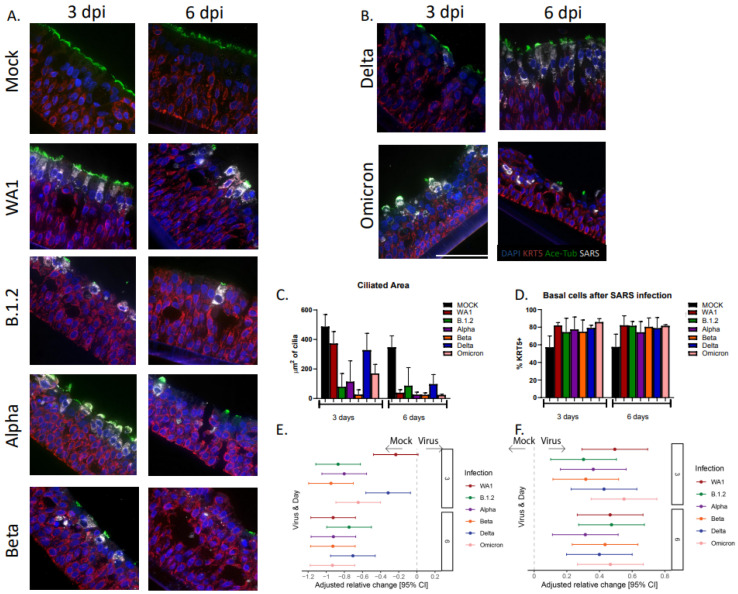
**Ciliary damage and percentage of basal cells in HNO-ALIs infected with strains of SARS-CoV-2.** (**A**,**B**) Representative IF imaging of a single adult HNO at 3 and 6 days post-infection (dpi) with six strains of SARS-CoV-2. Basal cells are stained in red by Krt5, ciliated cells are stained in green by acetylated alpha tubulin, SARS-CoV-2 viral particles are stained in gray by anti-spike protein antibody, and cellular nuclei are stained in blue by DAPI. Scale bar is 100 µm. (**C**) Area of ciliated epithelium in HNO-ALIs infected with strains of SARS-CoV-2 at 3 or 6 dpi. Error bars (standard deviation) represent two technical replicates from two donor HNO-ALIs. (**D**) Percentage of basal cells (number of Krt5 positive cells/ total cells) in HNO-ALIs infected with strains of SARS-CoV-2 at 3 or 6 dpi. Error bars (standard deviation) represent two technical replicates from two donor HNO-ALIs. (**E**) Forest plot of mock versus cilia area by virus strain modeled on the following factors: cell line, infection condition, and dpi. Each strain was compared against mock. Adjusted risk ratio estimates and their associated 95% confidence intervals are represented by dots and T-bars, respectively. (**F**) Forest plot of mock versus percentage of basal cells by viral strain was modeled on the following factors: cell line, infection condition, and dpi. Each strain was compared against mock. Adjusted risk ratio estimates and their associated 95% confidence intervals are represented by dots and T-bars, respectively.

**Figure 6:**
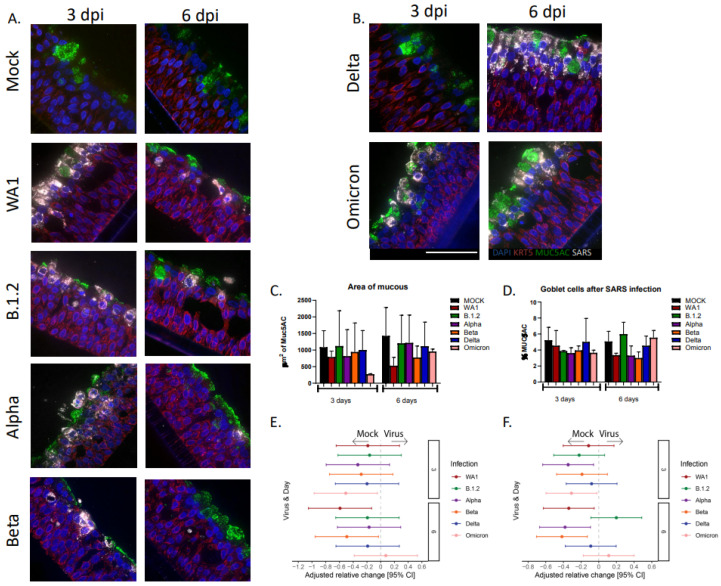
**Mucous secretion in HNO-ALIs infected with six strains of SARS-CoV-2.** (**A**,**B**) Representative IF imaging of a single adult HNO at 3 and 6 days post-infection (dpi) with six strains of SARS-CoV-2. Basal cells are stained in red by Krt5, goblet cells are stained in green by Muc5AC, SARS-CoV-2 viral particles are stained in gray by anti-spike protein antibody, and cellular nuclei are stained in blue by DAPI. Scale bar is 100 µm. (**C**) The area of mucus (Muc5AC+ area) in HNO-ALIs infected with strains of SARS-CoV-2 at 3 or 6 dpi. Error bars (standard deviation) represent two technical replicates from two donor HNO-ALIs. (**D**) Percentage of goblet cells (number of Muc5AC positive cells/ total cells) in HNO-ALIs infected with strains of SARS-CoV-2 at 3 or 6 dpi. Error bars (standard deviation) represent two technical replicates from two donor HNO-ALIs. (**E**) Forest plot of mock versus mucous area by virus strain modeled on the following factors: cell line, infection condition, and dpi. Each strain was compared against mock. Adjusted risk ratio estimates and their associated 95% confidence intervals are represented by dots and T-bars, respectively. (**F**) Forest plot of mock versus percentage of goblet cells (Muc5AC+ cells with DAPI+ nuclei over total number of DAPI+ cells) by viral strain was modeled on the following factors: cell line, infection condition, and dpi. Each strain was compared against mock. Adjusted risk ratio estimates and their associated 95% confidence intervals are represented by dots and T-bars, respectively.

An immunofluorescence quantification for apical ciliated cells, goblet cells, basal cells, and SARS-CoV-2 localization was performed during mid and late infection (3 and 6 dpi) to examine cell-type-specific changes in HNO-ALIs during SARS-CoV-2 infection. The virus was detected mostly in apical ciliated cells (Figure 5A,B), although rarely SARS-CoV-2 was found in goblet and basal cells (Figure 6A,B and File S1). All strains had significant ciliary damage demonstrated by the loss of acetylated tubulin immunostaining, which was more pronounced in the late infection. Interestingly, the WA1 and Delta strains of SARS-CoV-2 had reduced ciliary damage at 3 dpi compared to the other strains (Figure 5C).

We generated a relative risk ratio of apical ciliary damage compared to the mock infection using regression models of the raw area of the ciliated epithelium. At 3 dpi, all strains except WA1 had significantly less cilia. While still having significant damage, the Delta strain was the second-least damaging (Figure 5E). Conversely, when examining the basal stem cells, there was a significant increase in the basal cell population after infection with all variants (Figure 5D,E), potentially due to tissue cell repair during cellular loss of the virus infected apical ciliated cells, the most apical cell layer of the epithelium (Figure 5F).

### 3.4. Goblet Cells and Mucous Decrease After Infections with SARS-CoV-2 Variants

Goblet cells are responsible for the majority of mucus production in the lungs, and several respiratory pathogens have been shown to increase mucus production. To assess the impact of SARS-CoV-2 infection on mucus production, two parameters were evaluated, the amount of area staining for mucous and the percentage of mucous-producing goblet cells, both measured by Muc5AC. Largely, mucus production and goblet cell numbers did not significantly increase with infection (Figure 6A–D) and were comparable to the mock after infection with SARS-CoV-2 variants, except for strains WA1 that had significantly decreased areas of mucus at 6 dpi and Omicron at 3 dpi (Figure 6E). This was accompanied by a decrease in the percentage of goblet cells (Muc5ac staining with DAPI+ nuclei/total number of DAPI+ cells), which was statistically significant at 3 dpi for the Alpha and Omicron strains and at 6 dpi for the WA1, Alpha, and Beta strains (Figure 6F).

## 4. Discussion

We sought to examine how six common circulating variants of SARS-CoV-2 during the pandemic altered the respiratory epithelium in an ex vivo human challenge model of the HNO-ALI system. Importantly, these HNO-ALIs, derived non-invasively from tissue stem cells of the adult human nose, were obtained prior to the development of COVID vaccines, and thus represent an ideal HNO-ALI model for comparing one variant to another. Our study revealed distinct pathobiological differences that may explain variant-specific clinical presentations and epidemiological patterns observed during the COVID-19 pandemic. These findings add to the existing literature on our understanding of how viral mutations influence host–pathogen interactions at the nasal epithelium.

The delayed replication kinetics observed with the Delta variant—achieving a steady state later than other variants—suggests a unique viral strategy that may have contributed to its epidemiological success. This “stealth” replication pattern potentially allowed Delta to establish infection in the nasal epithelium before triggering robust immune responses, consistent with the clinical observations of delayed symptom onset in Delta infections [36]. Similar variant-specific replication differences have been reported in other nasal and airway organoid systems [17,37,38]. Chiu et al. reported that, in both 3D nasal organoids and monolayers, the Delta variant had higher viral loads than the SARS-CoV-2 wildtype strain (similar to WA1 in our study); however, the viral titers were lower than Omicron [17]. Another study by Hysenaj et al. on lower airway organoids showed a decreased stepwise replication of WA1 to Delta and to Omicron [37]. More recently, Tanneti et al. demonstrated that, in primary human epithelial cultures, Omicron was the fastest replicating virus as compared to Delta and WA1 [38]. In addition, Meganack et al. found that Delta displayed distinct replication patterns in lung airway epithelial cells, while Suzuki et al. demonstrated variant-specific differences in replication sites, with Omicron preferring upper airway tissues [21,39]. The differences in viral loads observed in our study and across various studies may be attributed to inherent genetic variations in the donors of the organoids or cells, as well as to the specific viral variant strains used in our study and others.

Cytokine induction after infection with SARS-CoV-2 variants was similar to that seen in patients with COVID-19, with an increase predominantly in pro-inflammatory cytokines and chemokines. IL-6, IL-8, and IP-10 were induced after infection and have been associated with worse outcomes in COVID infection [40,41,42,43,44,45,46]. In contrast to other variants, Delta elicited a somewhat dampened cytokine response, specifically in the pro-inflammatory cytokines and chemokines. Interestingly, this replicates a clinical study showing that Delta induces a less robust cytokine response than other variants [47]. Furthermore, Delta did not induce IL-6, CXCL9, IP-10, or CXCL11 like other variants, all of which have been detected in high levels in patients with COVID [11,30,40,41,43,44,45]. This observation may also be attributed to the fact that our HNO-ALI is a model of the upper respiratory tract (nose), whereas an infection in the lower respiratory tract (bronchial tree and alveoli) could elicit a distinct cytokine and chemokine response profile. The immune evasion capability of Delta may have contributed to its enhanced pathogenicity reported in unvaccinated individuals [48]. Additionally, Saito et al. demonstrated that Delta harbors mutations that antagonize interferon signaling more effectively than previous variants, supporting our observation of dampened innate immune responses [49]. The robust inflammatory signatures observed with WA1, Alpha, Beta, and Omicron align with previous studies showing that these variants trigger strong NF-κB-mediated inflammatory responses in respiratory epithelia [50,51]. Interestingly, our finding that TGF-β was significantly elevated early in Delta and Omicron infections suggests an additional immunomodulatory mechanism not previously appreciated with these variants, potentially contributing to dysregulated tissue repair [52].

All variants of SARS-CoV-2 potently decreased the overall area of cilia, similar to multiple other studies [17,53]. Our observation that WA1 and Delta caused less ciliary damage at 3 dpi compared to other variants provides important insights into how variants differentially impact respiratory epithelial integrity. This finding is consistent with studies by Hui et al., who reported variant-specific differences in ciliary damage in bronchial organoids [19]. In contrast to our study, infection with the Delta variant in primary nasal cultures resulted in a compromise of cell barrier integrity and a loss of nasal cilia and ciliary beating function [38]. In our HNO-ALI cultures, the relatively preserved ciliary architecture with the Delta infection early in the infection, despite viral replication, suggests that Delta’s pathogenicity may derive less from direct cytopathic effects and more from its ability to evade immune surveillance while establishing a persistent infection. While ciliated cells appeared to be the primary target for SARS-CoV-2 [54,55], we did detect rare mucus-producing goblet cells that were infected with SARS-CoV-2 [56], as well as basal stem cells. This finding is especially exciting in the subtext of long COVID and lung remodeling.

A limitation of our study is the relatively small number of HNO-ALI donor lines evaluated. Expanding the cohort in future studies would help to strengthen and validate our findings. Additionally, it is important to note that the HNO-ALI system models the nasal epithelium, and therefore the observations reported here may not fully reflect responses occurring in the bronchioles and alveoli of the lower respiratory tract. Another limitation of our HNO-ALIs is the lack of immune cells, which clearly play a role in the overall cytokine release and cellular injury. This likely accounts for our lack of a significant upregulation of TNFα, highly upregulated in clinical COVID infections [57]. However, the lack of immune cells allows for an isolation of the innate epithelial immune response, as well as virus-mediated cellular damage. Future studies which incorporate co-culture systems with immune cells to better recapitulate the complex host–pathogen interactions during SARS-CoV-2 infection will provide further strain-specific responses. Another limitation is that newer SARS-CoV-2 variants that emerged during the post-pandemic period were not included in our study. However, these six early variants provide an overall picture of the epithelial innate immune response and airway cellular injury that new emerging variants could cause.

Together, our data suggests that the Delta variant could act as a “stealth virus,” capable of evading the innate epithelial immune response of the nose. This immune evasion could allow it more time to invade the lower respiratory tract of the host. While the idea of immune evasiveness in COVID-19 infection is not new, typically Delta and Omicron are discussed together, with Omicron being the most evasive. Our comparative analysis of six early SARS-CoV-2 variants in HNO-ALIs reveals that different variants employ distinct strategies of replication and immune evasion, potentially explaining the enhanced transmissibility and virulence observed during the pandemic, and could potentially be applied to new variants arising post-pandemic. These findings highlight the value of the HNO-ALI system in understanding variant-specific pathobiology. Furthermore, this approach could serve as a valuable platform for the rapid assessment of emerging variants, providing early insights into their potential clinical and epidemiological impact.

## Data Availability

A Supporting Data Values file with all reported data values will be available as part of the Supplementary Materials.

## Supplementary Materials

The following supporting information can be downloaded at https://www.mdpi.com/article/10.3390/v17101343/s1. File S1: Supplementary Figures; File S2: Supplementary Data.
