# Supplementary figures and images for: Strain-Specific Variability in Viral Kinetics, Cytokine Response, and Cellular Damage in Air–Liquid Cultures of Human Nasal Organoids After Infection with SARS-CoV-2
Source: Viruses. 2025 Oct 6;17(10):1343. doi: 10.3390/v17101343 (PMC12567804; doi:10.3390/v17101343)

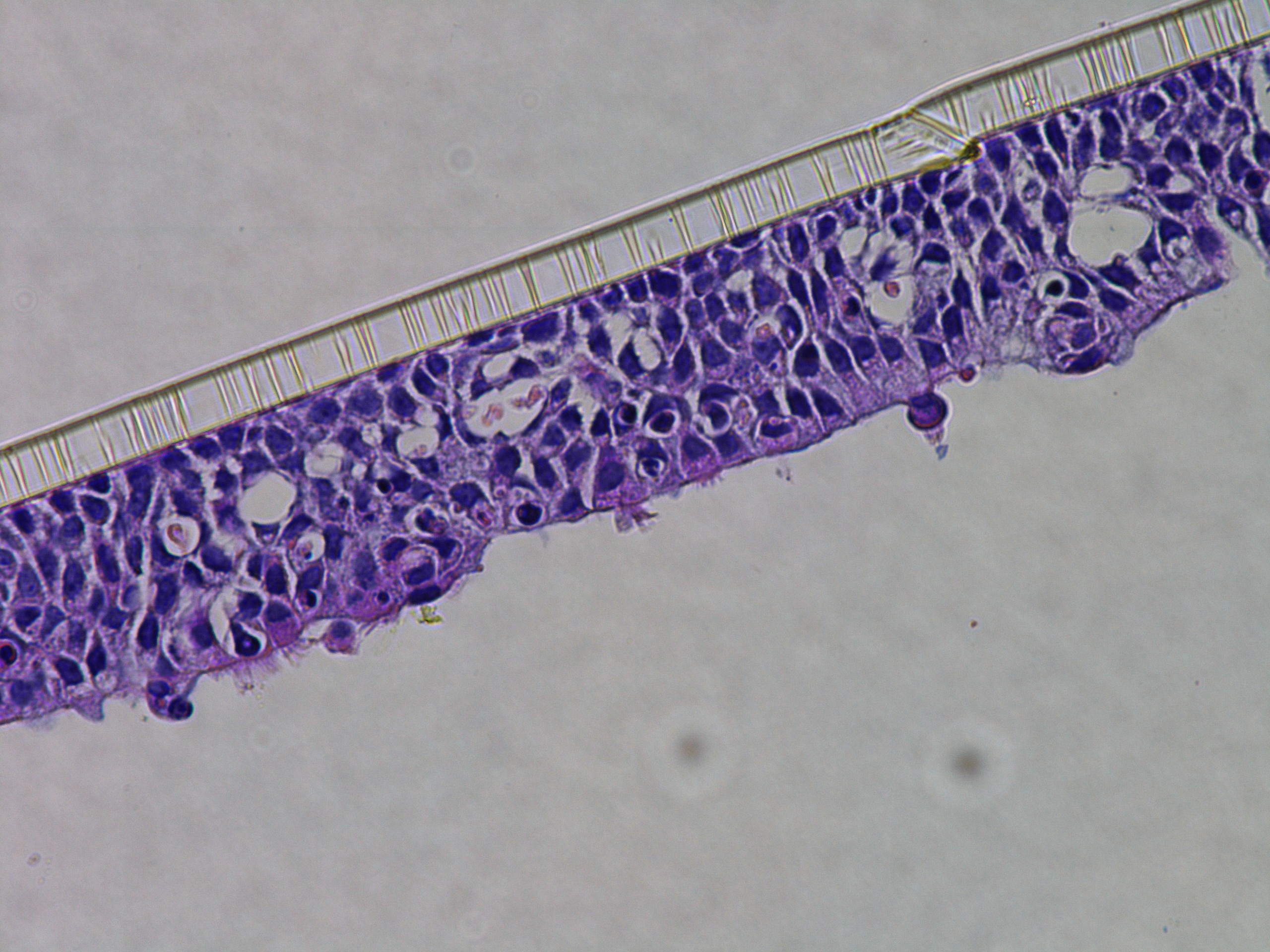

Supplement: Supplementary file 1 [file viruses-17-01343-s001.zip › File S1/Alpha day 3.tif]

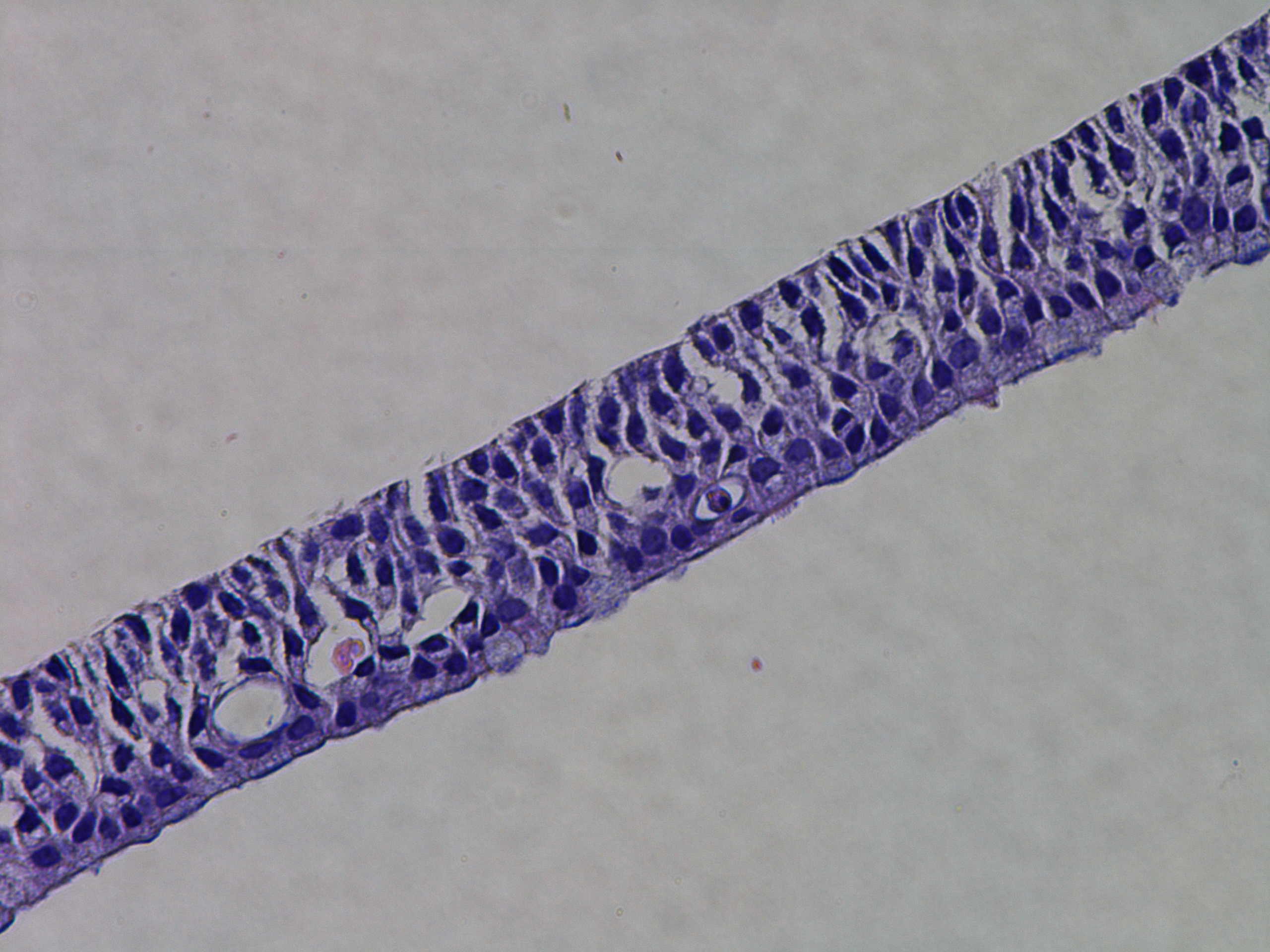

Supplement: Supplementary file 1 [file viruses-17-01343-s001.zip › File S1/Alpha day 6.tif]

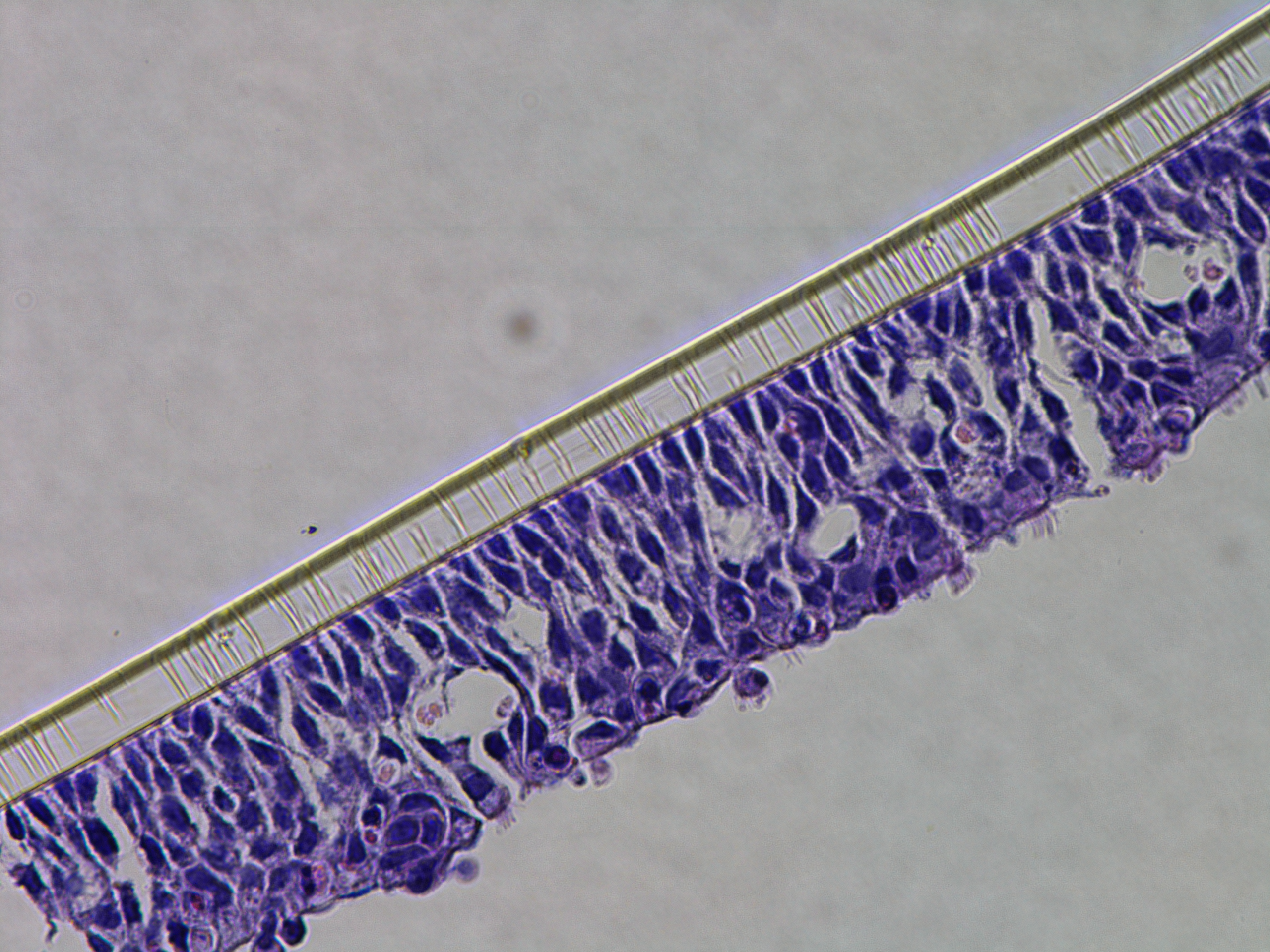

Supplement: Supplementary file 1 [file viruses-17-01343-s001.zip › File S1/B1.2 day 3.tif]

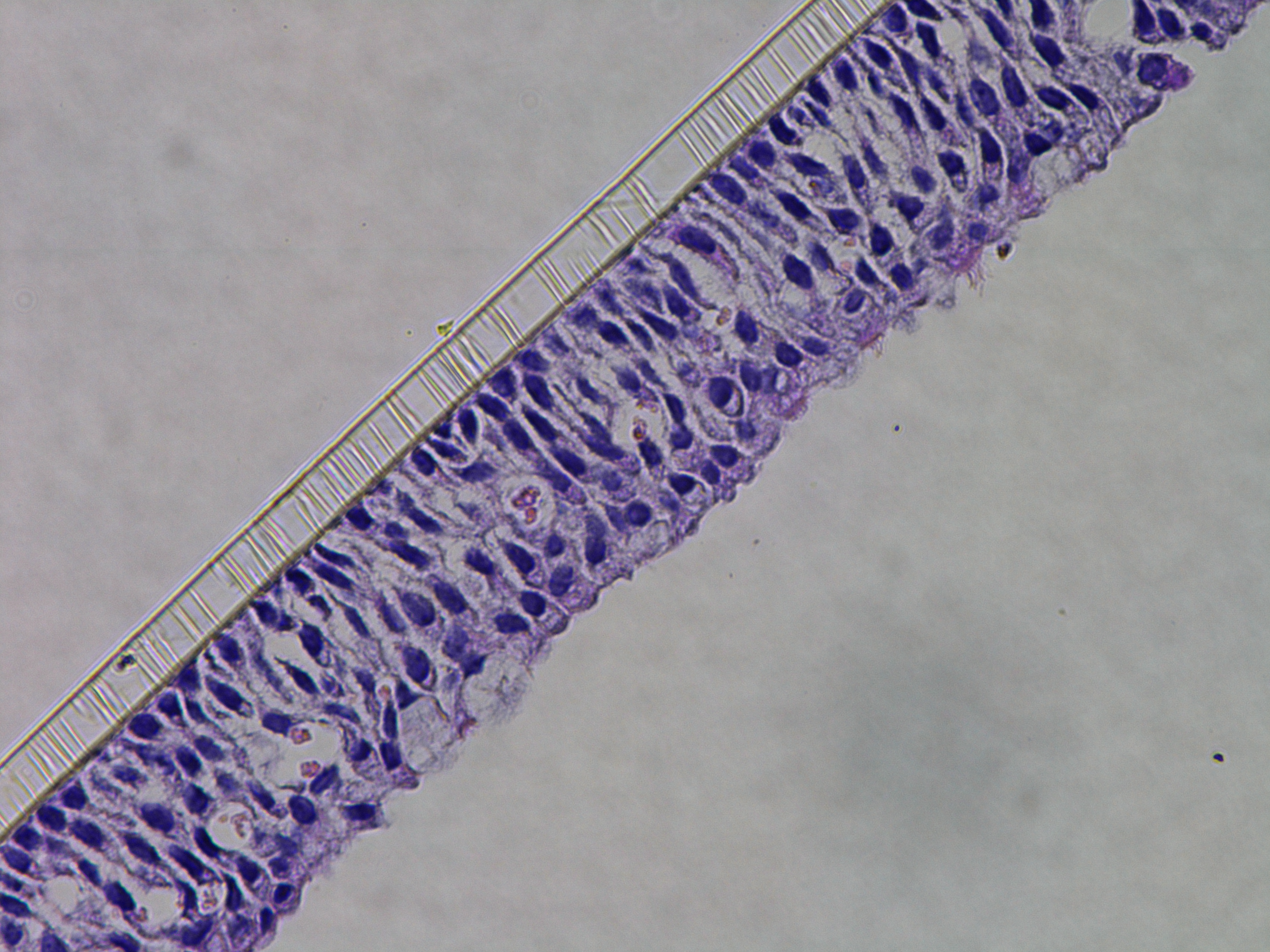

Supplement: Supplementary file 1 [file viruses-17-01343-s001.zip › File S1/B1.2 day 6.tif]

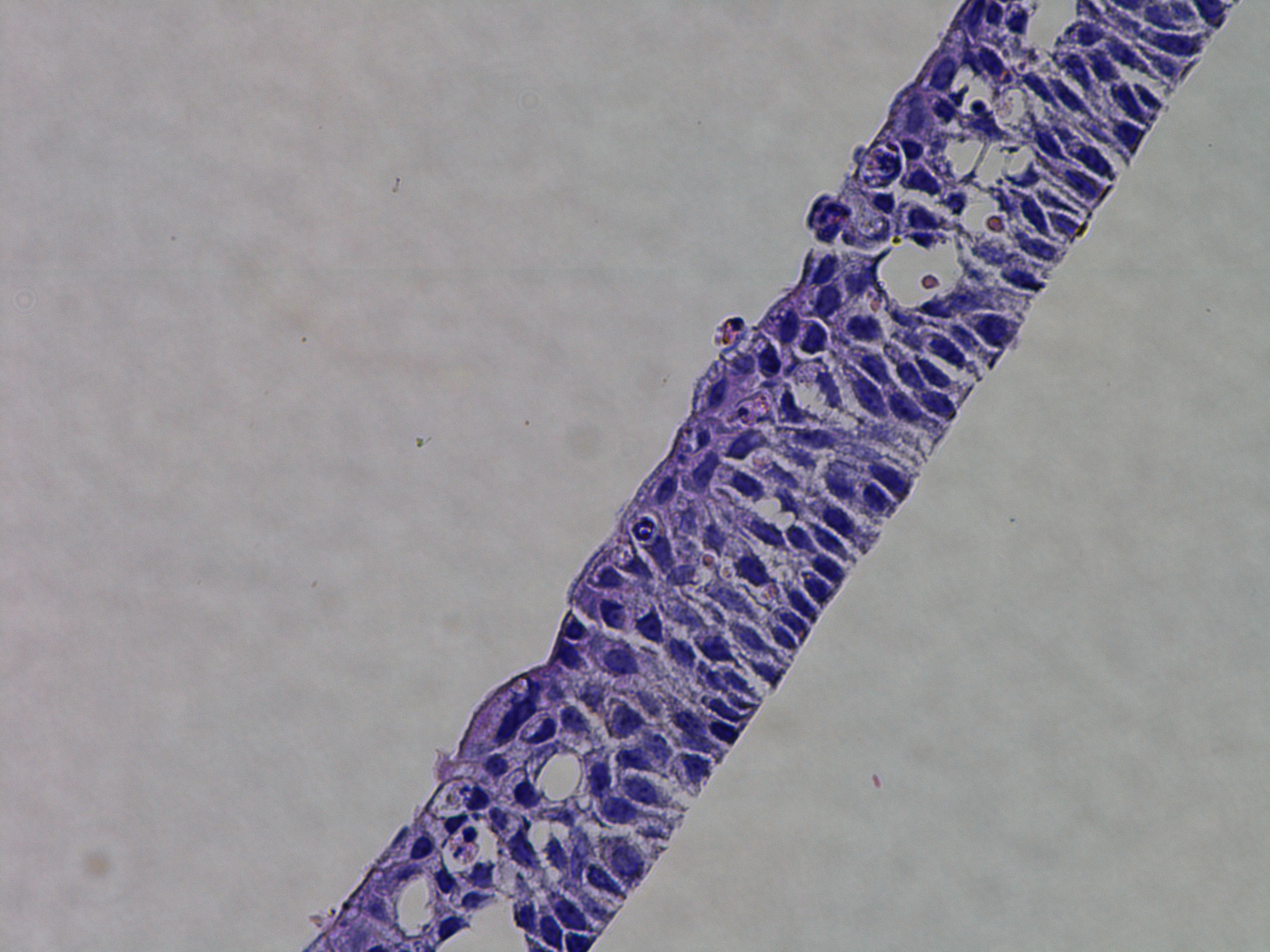

Supplement: Supplementary file 1 [file viruses-17-01343-s001.zip › File S1/Beta day 3.tif]

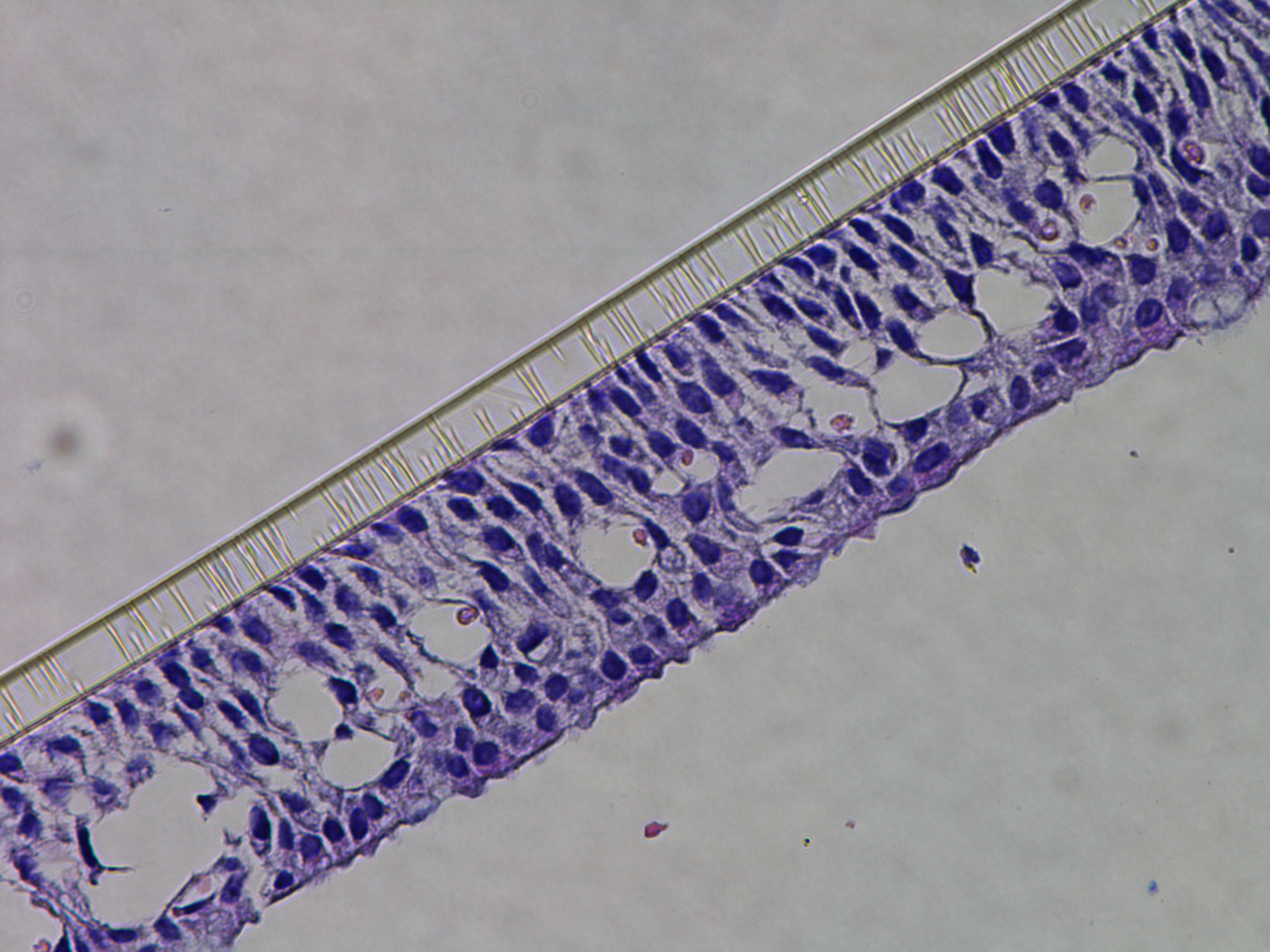

Supplement: Supplementary file 1 [file viruses-17-01343-s001.zip › File S1/Beta day 6.tif]

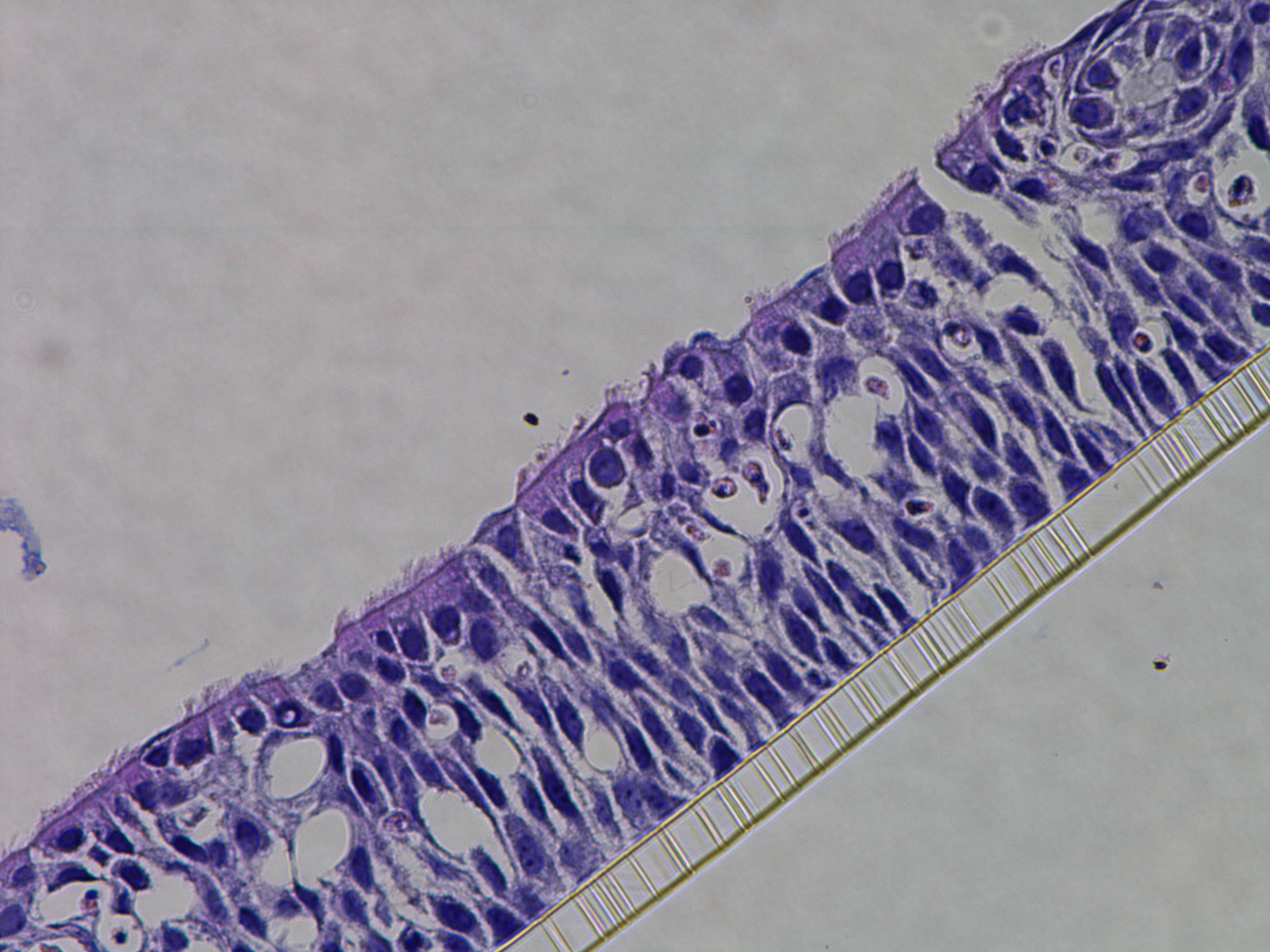

Supplement: Supplementary file 1 [file viruses-17-01343-s001.zip › File S1/Delta day 3.tif]

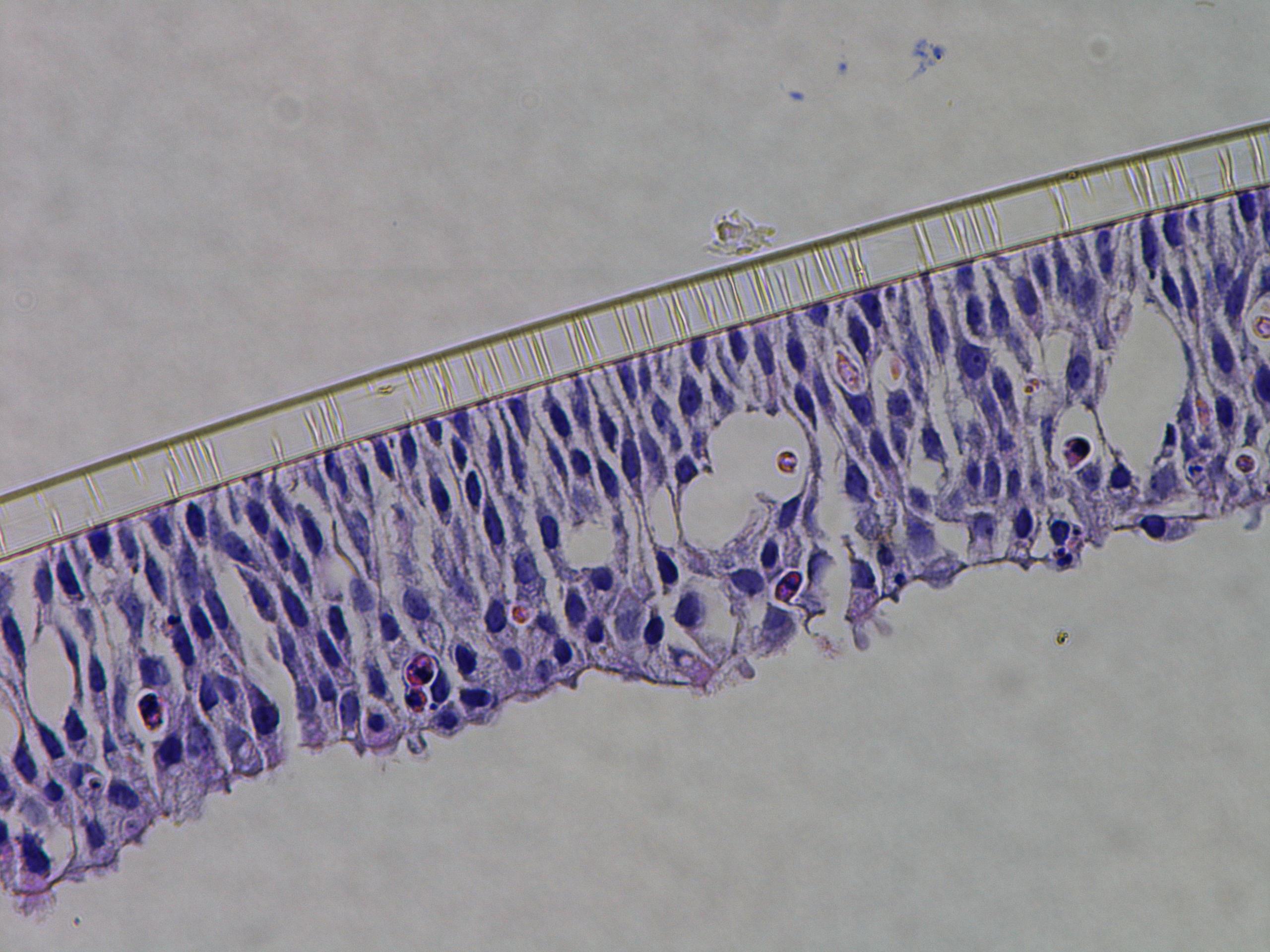

Supplement: Supplementary file 1 [file viruses-17-01343-s001.zip › File S1/Delta day 6.tif]

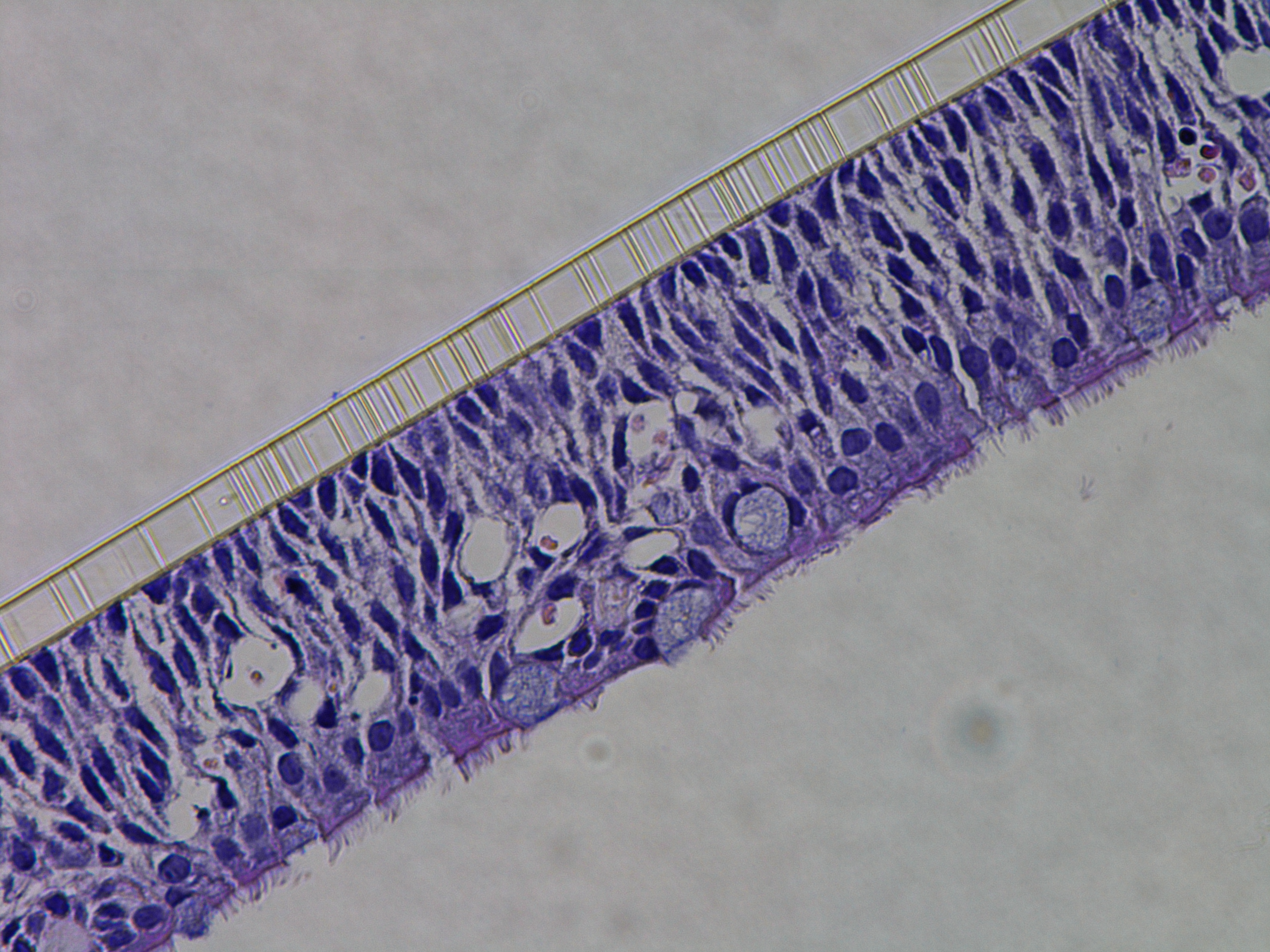

Supplement: Supplementary file 1 [file viruses-17-01343-s001.zip › File S1/Mock day 3.tif]

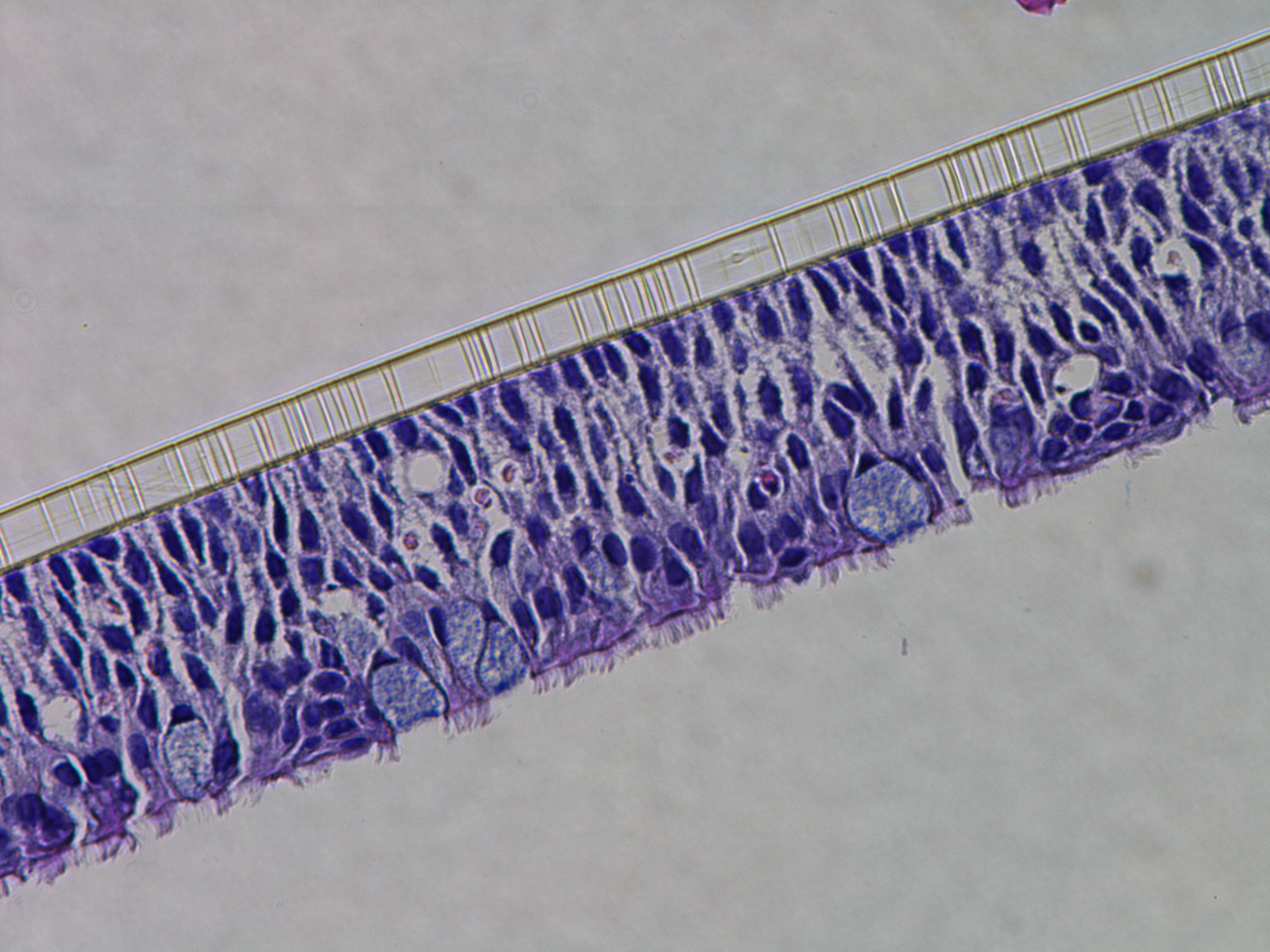

Supplement: Supplementary file 1 [file viruses-17-01343-s001.zip › File S1/Mock day 6.tif]

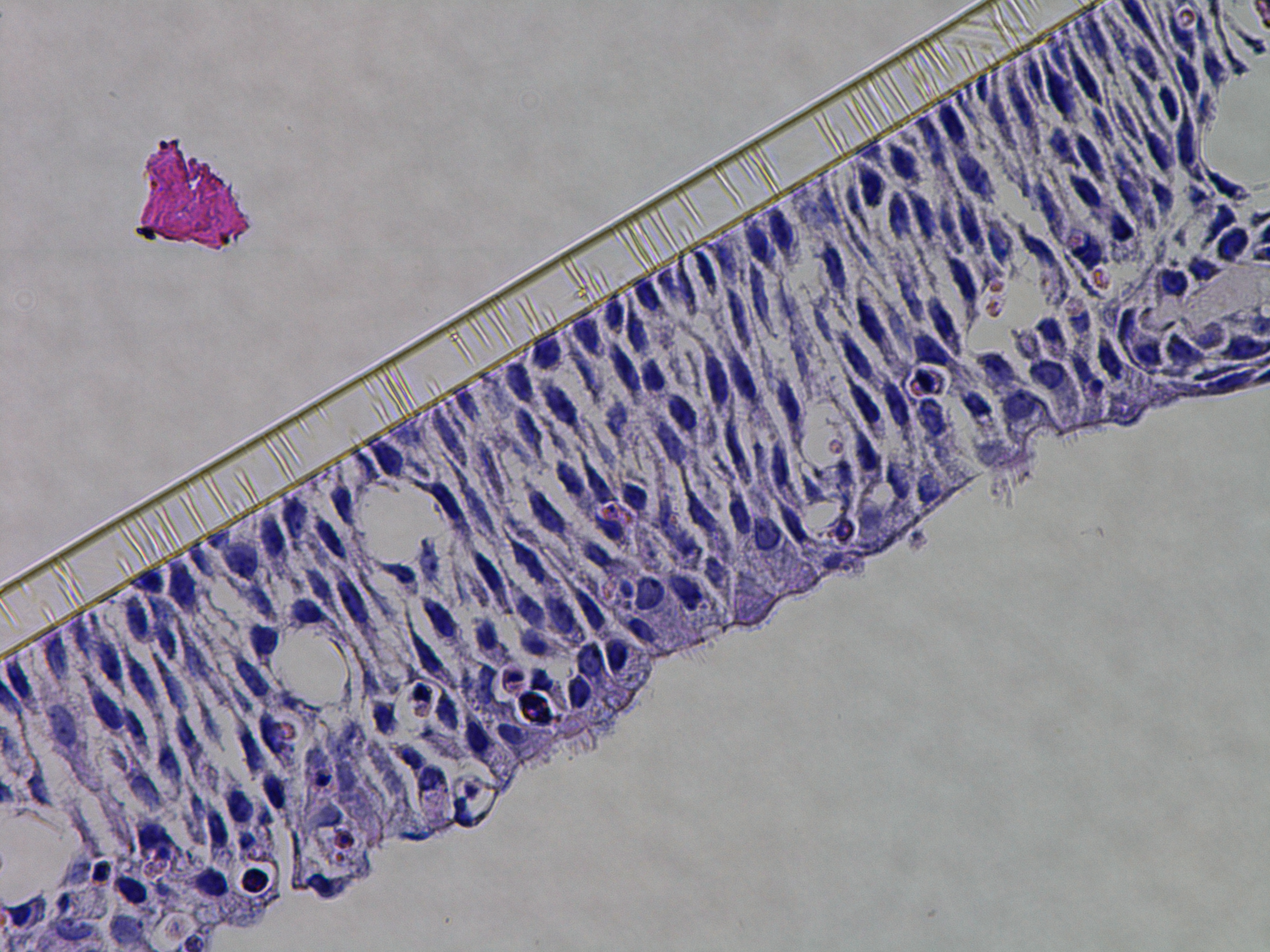

Supplement: Supplementary file 1 [file viruses-17-01343-s001.zip › File S1/Omicron day 3.tif]

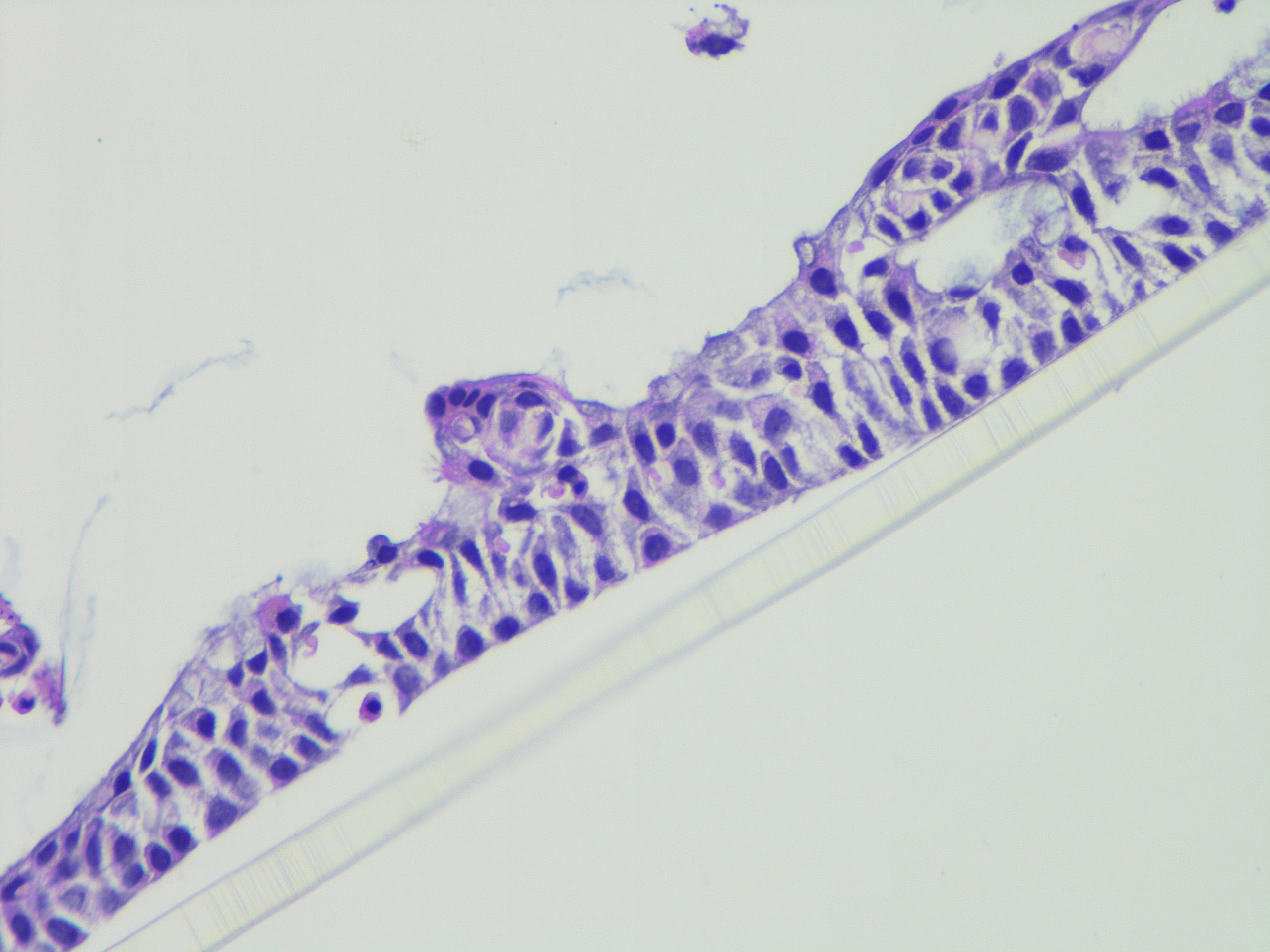

Supplement: Supplementary file 1 [file viruses-17-01343-s001.zip › File S1/Omicron day 6.tif]

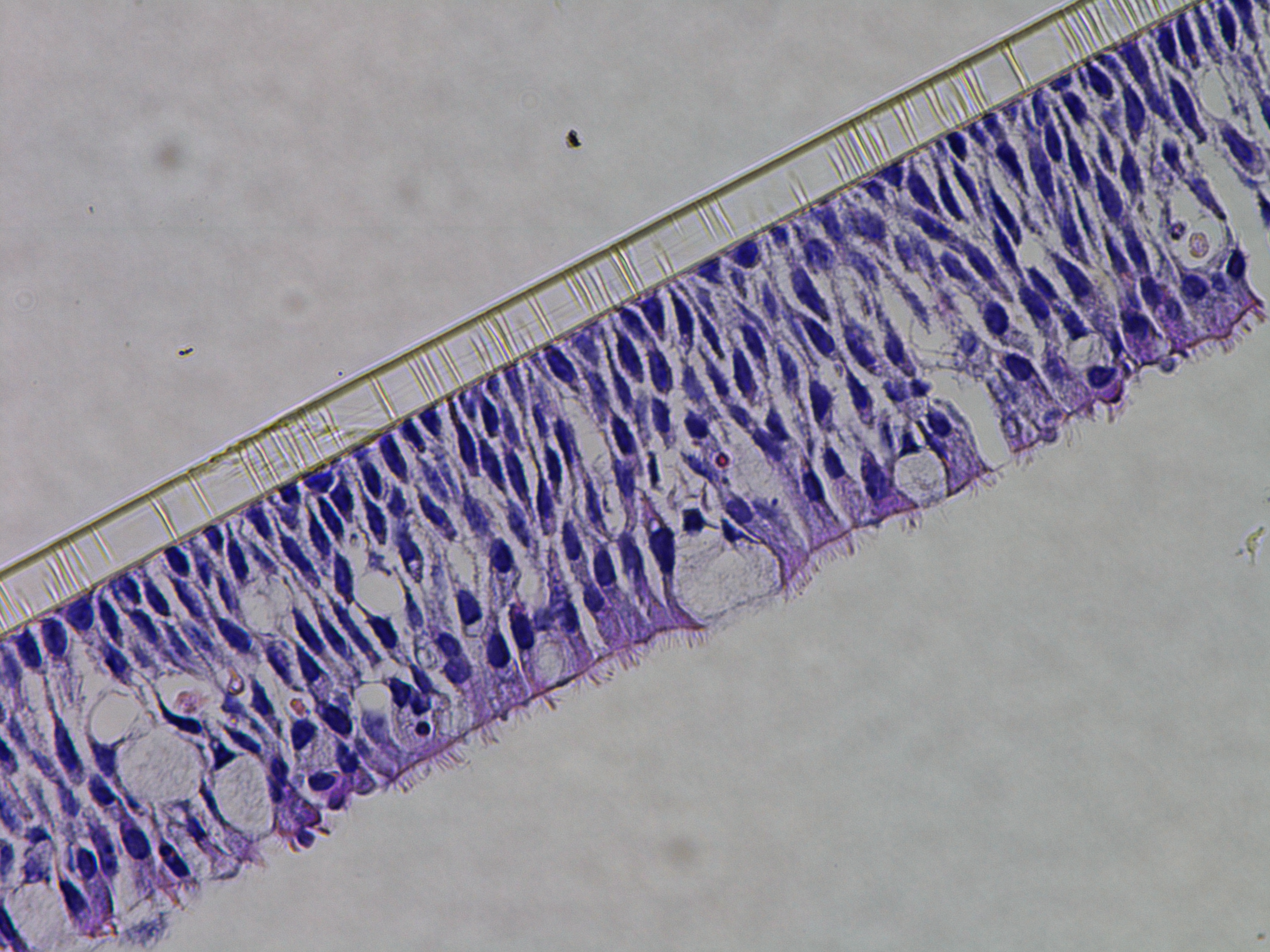

Supplement: Supplementary file 1 [file viruses-17-01343-s001.zip › File S1/WA day 3.tif]

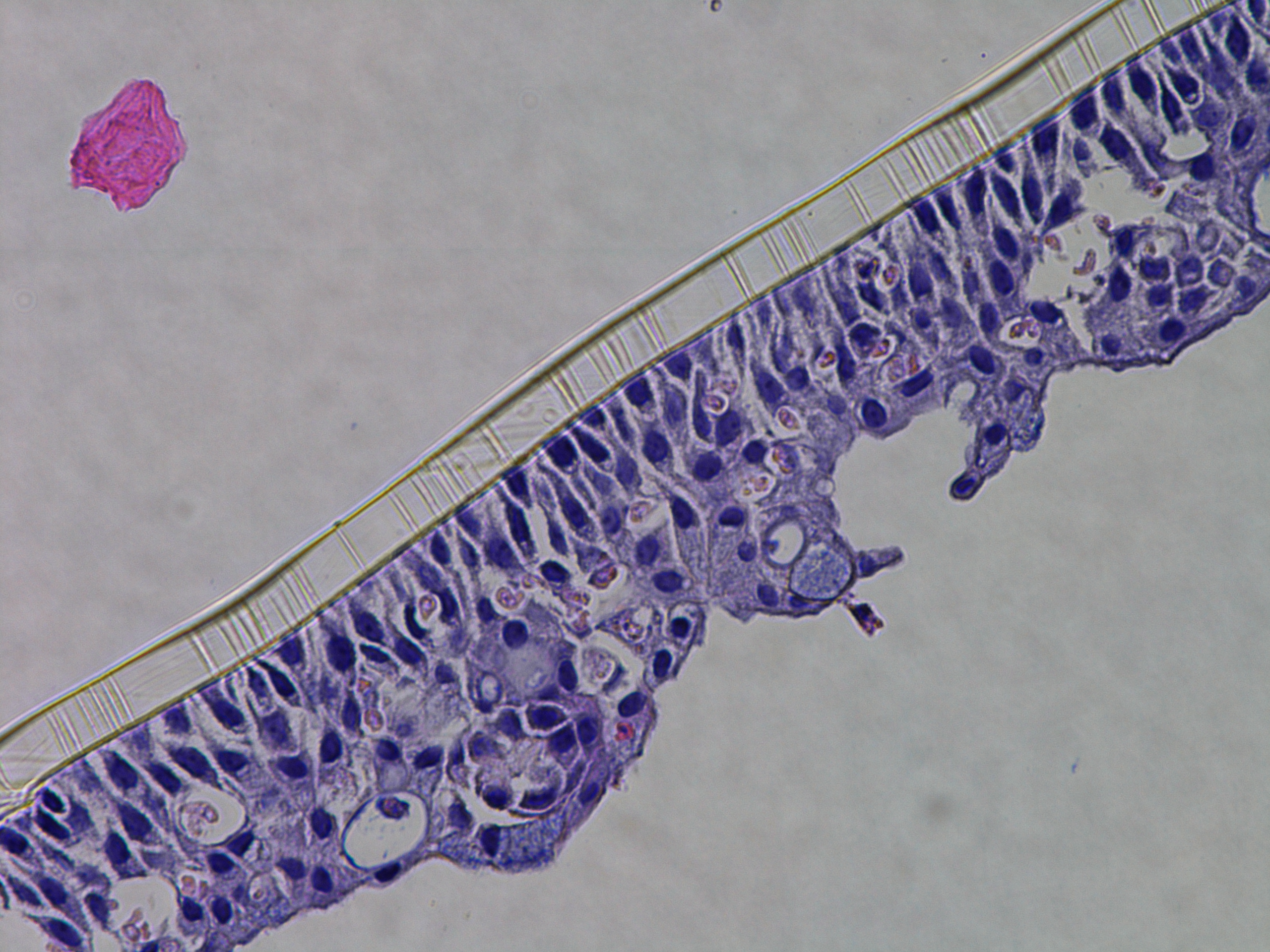

Supplement: Supplementary file 1 [file viruses-17-01343-s001.zip › File S1/WA day 6.tif]
